# Perfusion and apparent oxygenation in the human placenta (PERFOX)

**DOI:** 10.1002/mrm.27950

**Published:** 2019-08-21

**Authors:** Jana Hutter, Anita A. Harteveld, Laurence H. Jackson, Suzanne Franklin, Clemens Bos, Matthias J. P. van Osch, Jonathan O'Muircheartaigh, Alison Ho, Lucy Chappell, Joseph V. Hajnal, Mary Rutherford, Enrico De Vita

**Affiliations:** ^1^ Centre for the Developing Brain King's College London London United Kingdom; ^2^ School of Medical Engineering King's College London London United Kingdom; ^3^ Department of Radiology University Medical Center Utrecht Utrecht The Netherlands; ^4^ C.J. Gorter Center for High Field MRI Department of Radiology Leiden University Medical Center Leiden The Netherlands; ^5^ Academic Women's Health Department King's College London London United Kingdom

**Keywords:** Arterial Spin Labeling (ASL), perfusion, placenta, pre‐eclampsia, relaxometry, velocity‐selective ASL

## Abstract

**Purpose:**

To study placental function—both perfusion and an oxygenation surrogate ($T_{2}^{*}$)—simultaneously and quantitatively in‐vivo.

**Methods:**

Fifteen pregnant women were scanned on a 3T MR scanner. For perfusion measurements, a velocity selective arterial spin labeling preparation module was placed before a multi‐echo gradient echo EPI readout to integrate $T_{2}^{*}$ and perfusion measurements in 1 joint perfusion‐oxygenation (PERFOX) acquisition. Joint motion correction and quantification were performed to evaluate changes in $T_{2}^{*}$ and perfusion over GA.

**Results:**

The optimized integrated PERFOX protocol and post‐processing allowed successful visualization and quantification of perfusion and $T_{2}^{*}$ in all subjects. Areas of high $T_{2}^{*}$ and high perfusion appear to correspond to placental sub‐units and show a systematic offset in location along the maternal‐fetal axis. The areas of highest perfusion are consistently closer to the maternal basal plate and the areas of highest $T_{2}^{*}$ closer to the fetal chorionic plate. Quantitative results show a strong negative correlation of gestational age with $T_{2}^{*}$ and weak negative correlation with perfusion.

**Conclusions:**

A strength of the joint sequence is that it provides truly simultaneous and co‐registered estimates of local $T_{2}^{*}$ and perfusion, however, to achieve this, the time per slice is prolonged compared to a perfusion only scan which can potentially limit coverage. The achieved interlocking can be particularly useful when quantifying transient physiological effects such as uterine contractions. PERFOX opens a new avenue to elucidate the relationship between maternal supply and oxygen uptake, both of which are central to placental function and dysfunction.

## 1 INTRODUCTION

**Figure 1:**
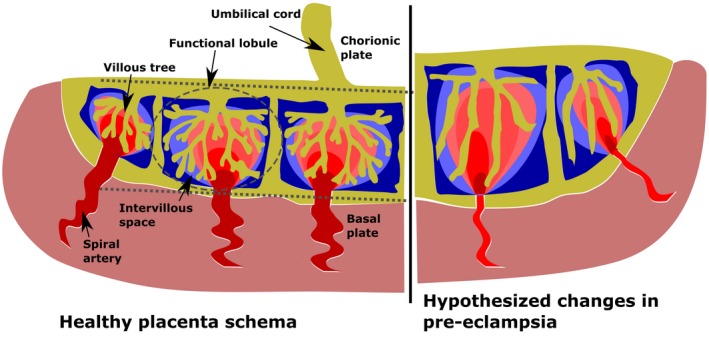
The placenta is depicted schematically. The spiral arteries ensuring supply from maternal side, the villous trees for oxygen uptake as well as a (schematic) depiction of the oxygen concentration from red (high oxygen content) to blue (low oxygen content) are illustrated together with the definition for a functional lobule used in the following. Furthermore, dotted lines point to the basal and chorionic plate. The right side shows some of the hypothesized changes in preeclampsia: elongated, less‐capillarized trees, and increased thickness

The human placenta constitutes the only link between mother and fetus. It supplies the fetus with oxygen and nutrients and ensures the elimination of waste products. It furthermore has essential endocrine and immunological functions. Major pregnancy complications such as pre‐eclampsia, fetal growth restriction, and preterm birth—all carrying a substantial risk of increased morbidity and mortality for both mother and child—are linked with placental insufficiency. Anatomically, the human placenta is composed of 10‐40 functional lobules.^1^ Each lobule contains 1‐2 maternal spiral arteries, suppling maternal blood from the uterine arteries into the inter‐villous space and thus irrigating these exchange units with oxygen‐rich blood (see Figure 1, left side). The fetal villi contain extensive arterial‐capillary‐venous systems originating from the umbilical cord, and are bathed in maternal blood. Special adaptions in early pregnancy include remodeling of the maternal spiral arteries, which allows for a slow and constant blood flow and thus ideal perfusion of the functional units by maximizing the contact area and the transfer time. Ongoing maturation across gestation leads to denser, more capillarized vascular trees and decreasing thickness of the cellular layer which separates villi and maternal blood.

Placental insufficiency is linked to smaller than normal vascular lumen of the maternal spiral arteries as a sign of incomplete remodeling. Furthermore, less capillarized, elongated villi can be observed^1^ and are schematically depicted in Figure 1 (right side). These structural findings are generally characterized ex vivo using histopathology. While these insights are valuable, they do not directly inform on causality or the cascade of events that might link different structural features. This requires imaging the placenta in vivo, which is currently mostly performed using ultrasound (US). However, current US screening tends to focus mainly on flow measurements in the umbilical cord and uterine artery and thus fails to provide direct insight into the placental functional core. This lack of a suitable in vivo observation window hampers early diagnosis and prevents understanding of the complex disease etiology.

A recent surge in placental MRI studies showed promising results to bridge this gap and allow in vivo assessment of placental function during gestation. The ability of MRI to generate contrasts adapted to microstructure and tissue properties renders this technique ideally suited to visualize the cascade of events within the placenta leading ultimately to placental insufficiency. Among these, perfusion measurements have been performed using a variety of techniques: Intravoxel incoherent motion (IVIM),^2^, ^3^, ^4^, ^5^, ^6^, ^7^, ^8^, ^9^, ^10^ arterial spin labeling (ASL),^11^, ^12^, ^13^, ^14^, ^15^ and lately, velocity‐selective arterial spin Labeling (VSASL)^16^, ^17^ have all been used. VSASL has the advantage that it does not require a geometrical separation of the blood labeling region and the perfusion observation region. It labels blood that is flowing at a speed above a user defined cutoff. The longitudinal magnetization of blood flowing above this cutoff is saturated. During a post‐label delay the tagged blood flows down the arterial system and modifies the magnetization in the imaging volume.

Relaxometry techniques has been successfully used for oxygenation studies of the placenta.^5^, ^18^, ^19^, ^20^, ^21^, ^22^ $T_{2}^{*}$ is of specific interest because there is a well‐established relationship between this parameter and oxygenation through the blood oxygen level dependent (BOLD).^23^ While it is reasonable to regard $T_{2}^{*}$ as an indicator of oxygen concentration it is not a direct measure. Important confounding factors include microstructural geometry effects, e.g. due to the random diffusion of water molecules around vessels which lead to a reduced BOLD signal around smaller vessels and differences in the oxygen‐hemoglobin dissociation curve between fetal and adult hemoglobin.

Despite the recent increase in available techniques and interest, current placental imaging studies are often limited by focusing on an individual contrast and a tendency to evaluate parameters averaged over the entire placental volume. Given the complex disease etiology, physiological placenta studies could benefit from multiparametric analyses, that can locally link, e.g. maternal perfusion to the microscopic structure of the villous tree and oxygen exchange. There are, however, 2 significant challenges complicating such described multi‐modal assessments in placental MRI: (a) maternal respiration and fetal bulk movements decrease internal consistency between data acquisitions separated in time (b) examination times need to be kept short to ensure maternal comfort. This study therefore proposes a multidimensional simultaneous integrated assessment of perfusion and oxygenation called PERFOX: combining 2 independent functional MRI techniques, $T_{2}^{*}$ relaxometry and VSASL, allows to study the interaction between maternal perfusion and $T_{2}^{*}$ as a marker fetal oxygen uptake. Quantitative and qualitative results from 15 placentas illustrate the dynamic joint spatial patterns of perfusion and oxygenation in vivo over gestational age.

## 2 METHODS

### 2.1 Experiments

The study was approved by the local IRB (Riverside Ethics Committee REC 14/LO/1457). A total of 15 pregnant women (median/range gestational age (GA) 28.9/21.9‐38.2 weeks) were included and scanned subsequent to informed consent, in the supine position^24^ on a clinical 3T Philips Achieva MRI scanner (Best, Netherlands) with a 32‐channel receiver coil. Safety and comfort of the mother was ensured: bespoke padding was designed to support the lower back, all women were asked to lie on the left side first to shift the weight of the pregnant uterus off the vena cava before slowly transitioning into supine position. Furthermore, life monitoring using constant pulse oximetry and blood pressure measurements were performed at 10‐min intervals, and the scanner operator maintained frequent verbal interaction with the women. The examination was split in 2 sessions of 30 min separated by a break to increase patient comfort. All the women scanned for this study tolerated the supine position well.

Each scan session started with initial calibration scans: a T_2_‐weighted 2D single shot Turbo Spin Echo sequence and B0 map were acquired in coronal orientation covering the whole uterus. These enabled both image‐based shimming^25^ targeted to the placental parenchyma and planning of the acquisition geometry for the functional acquisitions. The proposed PERFOX scan was acquired next.

### 2.2 PERFOX read‐out

Strategies to deal with motion is of key importance due to the high prevalence of breathing and fetal motion. The image acquisition was thus performed with single‐shot echo planar imaging (ssEPI) to freeze motion within each slice. To limit acoustic noise, the ssEPI sequence was constrained by imposing an echo spacing of 1 ms (i.e. read‐out frequency of 500 Hz), shown previously to minimize acoustic noise on our scanner.^26^ Acoustic noise measurements were performed using an MR‐compatible Optoacoustics fiber optic microphone (Optimic 1155, resolution of 0.1 dB) to verify that the acoustic noice was kept below 105 dB(A). The sensor was positioned at isocenter, the typical location of the fetal head, in the empty scanner bore to ensure stable measurement conditions. For most scans, the coronal slice orientation relative to maternal habitus was selected for maximal efficiency as it ensures that the longest dimension placentas located, as is most common, mainly anterior or posterior is parallel to the slice plane. An axial slice orientation was chosen for select acquisitions to better visualize the placenta from maternal to fetal side in 1 plane. Both in‐plane resolution and slice thickness were fixed to 4 mm.

### 2.3 Intrinsic contrast mechanisms

VSASL,^27^ implemented in a similar manner to^28^ was employed to visualize perfusion within the placental parenchyma. The sequence consists of a velocity‐selective tagging module, parametrized by the cutoff (*V_c_*) and a post‐label delay (PLD), a background suppression (BGS) module consisting of 2 inversion pulses, and the EPI read‐out module. As in conventional implementations of VSASL, the tagging module is spatially non‐selective. However, the gradients in the tagging module are applied along a chosen axis and only blood flowing in this direction is labeled. Labeling in the maternal superior‐inferior direction was judged most effective (largest blood signal change) in preliminary investigations irrespective of scan plane orientation, so this was adopted for all examinations. Acquisition of control images with the gradients in the tagging module set to zero and subtracting these from labeled images removes the static tissue signal contributions. Each pair of label and control images, acquired in interleaved order is referred to as one dynamic. A consequence of using a 2D multi‐slice acquisition in combination with VSASL is that each slice within the imaging volume is excited at a different time relative to the tagging module. Each slice thus has its own PLD and presents with different degrees of BGS.

**Figure 2:**
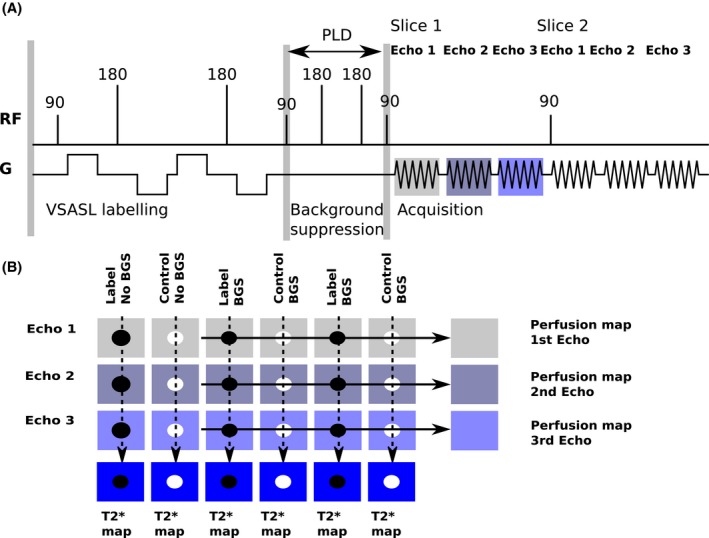
The acquisition strategy (A) and post‐processing (B) for PERFOX are depicted schematically. In (A) the VSASL labeling module, the background suppression module and the acquisition module—composed of 3 echoes—are depicted. NB. the horizontal time axis is not to scale

This basic VSASL sequence was modified and optimized to deliver information on perfusion and $T_{2}^{*}$ simultaneously by the addition of extra gradient echoes prescribed after the initial echo for each slice. This allows $T_{2}^{*}$ mapping independently for both label and control volumes as depicted in Figure 2A. This approach ensures a reduced sensitivity to motion, as all data required for $T_{2}^{*}$ fitting in each slice is acquired within <200 ms. The range of TEs for the multiple echoes was chosen based on placental $T_{2}^{*}$ values, obtained in a previous study (10‐150 ms).^5^

To assure that the perfusion results are comparable with respect to their position along the axis from maternal basal plate to fetal chorionic plate, the slice acquisition order was adjusted to be anterior‐to‐posterior for anterior placentas and posterior‐to‐anterior for posterior placentas.

Importantly, the inter‐slice PLD increment depends on the number of gradient echoes acquired, the higher the number of acquired echoes, the larger the PLD increment. The optimization of the joint scan required adjustment of the number of slices and the number of echoes, while ensuring that $T_{2}^{*}$ fitting was reliable and the coverage appropriate. Enforcing a similar maximal PLD for the last slice for PERFOX compared to a ‘separate’ VSASL acquisition resulted in 8 slices, compared to 13 slices (See Supporting Information Figure S1). The intrinsic link between inter‐echo spacing and length of the EPI train results in TEs of (20,56,93) ms for the standard PERFOX acquisition.

For the BGS, 3 preliminary T_1_ experiments made using ZEBRA^29^ allowed to determine a T_1_ range (900‐1200 ms) which was used to adjust sequence timings to make sure that all imaged slices had positive signal at the beginning of the read‐out of the first slice^30^; this resulted in the 2 inversion pulses being placed right after the tagging module and 1130 ms later. Supporting Information Figure S2 illustrates the effect of the choice of slice orientation on the acquired images, in terms of their differing dependence on PLD. The first dynamic is acquired without this background suppression module to provide a pseudo‐M0.

The imaging parameters for the coronal PERFOX scans were resolution 4^3^ mm^3^, Field‐of‐view 300 × 380 × 20 mm, 8 slices in ascending order, SENSE 2.5, Partial Fourier 0.97, TR = 3500 ms, PLD = 1600 ms, inter‐slice spacing 115 ms, 1 dynamic without BGS and 25 dynamics with BGS (pulse timings 50 ms and 1130 ms), Label 50 ms, *G* = 13 mT/m, *V_c_* = 2 cm/s, total acquisition time 3 min.

### 2.4 Post‐processing

Nonrigid motion correction was performed in ANTS.^31^ All VSASL volumes of the first echo time were registered to a common representative space created using an iterative template construction approach. After registering the volumes to an initial average of the VSASL dynamics, the template construction algorithm nonlinearly registered each volume to the template image and then constructed a representative shape image requiring the least transformation from all other volumes. This process was repeated but with the new representative image taking the place of the initial average in the registration. The parameters used for the nonlinear registration were the defaults for the script, using the Symmetric Normalization model, and a cost function with a voxel radius of 4. In a second step, the obtained transformations were employed to correct all volumes from subsequent echo times. This 2‐step registration approach follows the assumption that volumes acquired at the different subsequent TEs are aligned due to their temporal closeness (<200 ms) and do not require further registration. For quantitative whole‐organ results, the region of‐interest (ROI) was manually drawn on each slice of the first, non‐background‐suppressed control volume (i.e. the pseudo‐M0).

Once all volumes were aligned, perfusion analysis, and $T_{2}^{*}$ fitting were performed as depicted schematically in Figure 2B. For $T_{2}^{*}$ mapping, the signal values from all echoes were fitted voxel wise to a mono‐exponential decay curve $S\left(t\right)\;=\;S\left(0\right)exp^{\left(-T_{2}^{*}/TE\right)}.$ Using Levenberg‐Marquart optimization with initial parameters $T_{2}^{*}$ = 100 (ms) and *S*(0) = *S*(*TE*_1_). Both individual $T_{2}^{*}$ maps for each volume for control/tag volumes, respectively, and averaged $T_{2}^{*}$ maps for control and label volumes were calculated.

Perfusion analysis was performed by pairwise subtraction of the label‐control pairs with BGS. The resulting difference images were normalized by the pseudo‐M0. This normalization takes the unsaturated magnetization of the inflowing blood into account and removes the effect of the T_2_ weighting of the labeling module on the perfusion data; it therefore produces a semi‐quantitative quantity related to blood flow facilitating comparison of data from different subjects. This avoids absolute quantification which would require estimation or assumption of blood T_1_ and T_2_, since these are highly dependent on blood oxygenation, hematocrit and whether maternal or fetal blood is being considered. We therefore express the perfusion maps in arbitrary units. All displayed results—both the maps and the quantitative results are using these units. By averaging over multiple dynamics the cumulative result is obtained.

Quantitative evaluation was performed by averaging the values within the ROI to assess the relationship between $T_{2}^{*}$ and perfusion/M0 over GA. A one‐way ANCOVA was conducted to determine whether there is a statistically significant difference between placental location (anterior/posterior) on $T_{2}^{*}$ respectively on perfusion/M0 when controlling for gestational age.

All described analysis so far included the cumulative averaged subtraction results from all dynamics. The influence of the number of dynamics on the cumulative perfusion map was evaluated in a subgroup of participants using between 2 and all acquired dynamics for the quantification. The corresponding cumulative maps are displayed alongside each other together with plots from averaged signals from selected ROIs. In addition, a sliding window analysis was performed, whereby the perfusion map is the average over *l* subsequent dynamics, where *l* is the length of the sliding window.

### 2.5 Additional validation scans

Additional experiments were performed to evaluate parameter choice, robustness and versatility of PERFOX on a subset of subjects: (a) separate $T_{2}^{*}$ relaxometry, *n* = 5; separate VSASL, *n* = 5; and both separate $T_{2}^{*}$ and VSASL, *n* = 1. These separate scans were individually optimized for the respective purposes ‐ narrow range of PLD for VSASL over a number of slices *N_s_* and ideal spacing of echo times for $T_{2}^{*}$ measurements. Read‐out parameters such as resolution, echo spacing and TR were chosen to be as close as possible between these acquisitions and the PERFOX acquisition to enhance consistency. The $T_{2}^{*}$ acquisitions were performed in the same exam half immediately following the PERFOX scan; the VSASL scan was performed in the second exam half, after repositioning and new shimming. Reproducibility was studied in 3 volunteers where the PERFOX scan was repeated in the second half.

Finally, in 8 of 15 volunteers a PERFOX scan with higher resolution and axial slice orientation (PERFOX‐HIGH) was performed to explore the ability of the proposed PERFOX technique to visualize even finer details. The different orientation was chosen as discussed above, to allow visualization of the main functional axis of the placenta over a few select slices without the confounding effects of slice‐dependent PLD and BGS variation. The TEs were chosen as (28,83,137) ms.

## 3 RESULTS

**Figure 3:**
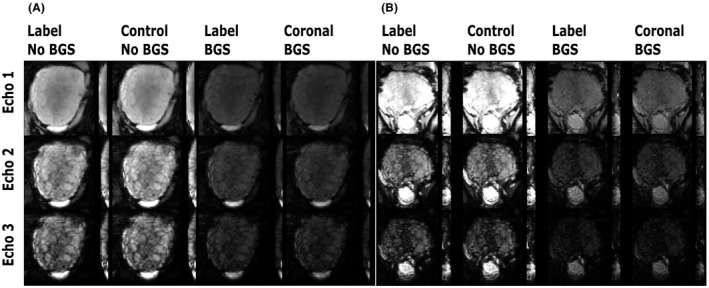
Depiction of 2 example PERFOX datasets from an (A) anterior and (B) posterior placenta. All volumes are shown in a mid‐parenchymal native coronal plane. Rows 1‐3 show images at TE1 to TE3

The joint PERFOX scan was successfully implemented and acquired on all participants: unprocessed images from 1 anterior placenta and 1 posterior placenta are shown in Figure 3.

### 3.1 Motion correction

Visual analysis of a subset of the initial datasets confirmed that the registration of consecutive echoes was not beneficial: the extensive $T_{2}^{*}$ differences between lobules and septa (tissue sections separating the lobules), result in different anatomical landmarks in the 3rd echo and frequent registration failures affecting also the alignment of the data from the shorter TEs. Better results were consistently achieved with the approach discussed above based on only estimating motion parameters from the volumes acquired at the first TE.

**Figure 4:**
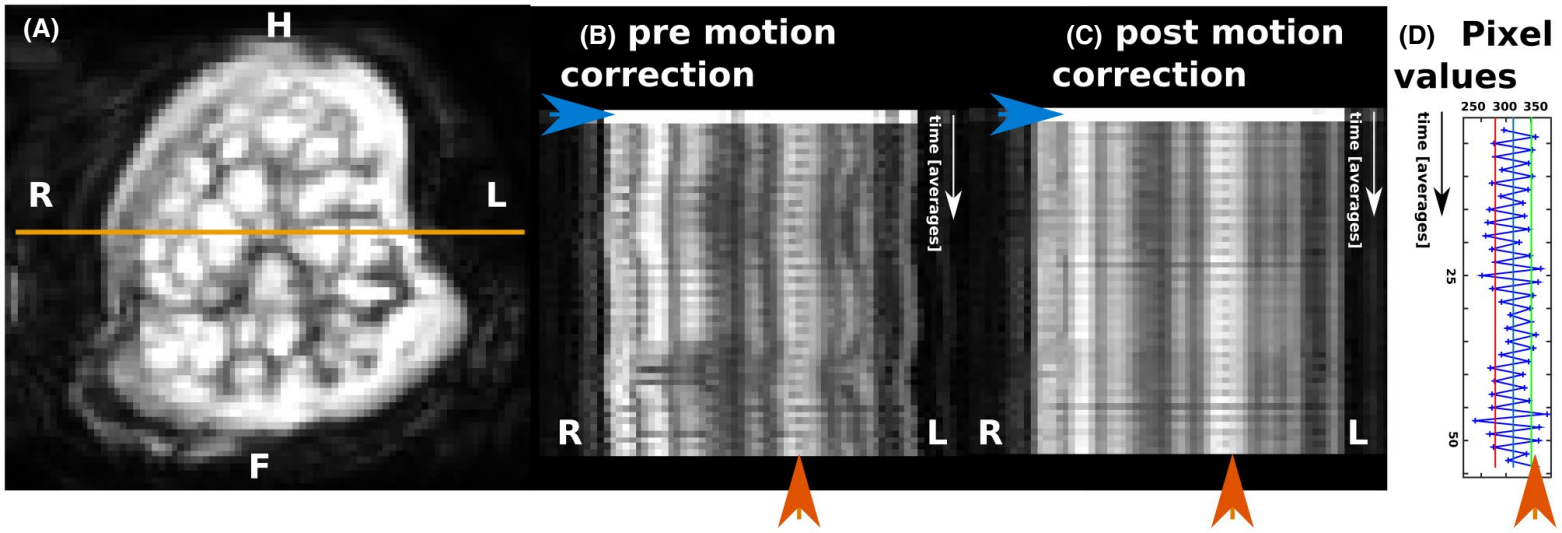
Illustration of the post‐processing motion correction results. In (A) one mid‐parenchymal slice in coronal orientation is shown to visualize the line in right‐left orientation which is shown (B) before and (C) after motion correction over all volumes (top to bottom). The blue arrows indicate the first non‐background suppressed volumes, the orange arrow highlights an area of high perfusion, where the interleaved contrast is seen clearly before and after motion correction. (D) Finally, the post motion correction signal at the voxel depicted by the orange arrow in (B) and (C) is plotted together with the mean of the control volumes (green) and the mean of the labeled volumes (red)

An example of the usefulness and efficacy of the motion correction is illustrated in Figure 4, depicting 1 L‐R line through the placenta for all dynamics (from top to bottom) pre‐ and post‐motion correction (B,C). A better alignment is observed after motion correction (C), depicting both the alignment of similar structures but also, shown by orange arrows, the consistent signal changes from label to control on 1 select area of high perfusion. The depicted signal across the ROI (is shown in (D) corresponding to a signal mean of 280 ± 17.08 for the labeled and 350 ± 12.36 for the control volumes (compared to 305 ± 31.02 and 360 ± 30.45 pre‐motion correction)—thus allowing for clear determination of the control‐label signal difference.

**Figure 5:**
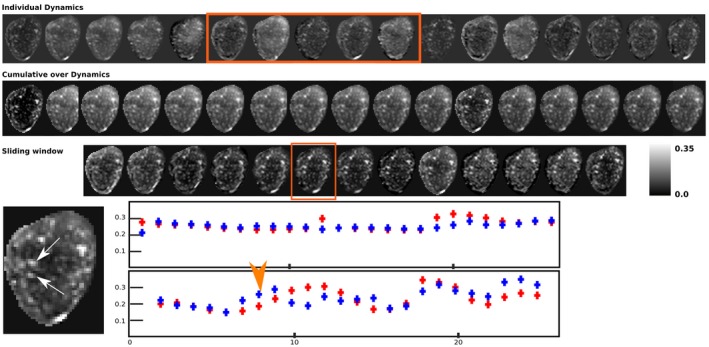
Results over dynamics. All dynamics are shown after subtraction in the top row, the results from cumulative analysis in the middle and the sliding window results with a window length of *l* = 5 in the bottom row. Finally, quantitative results from 2 voxels (red and blue) in high perfusion areas are depicted as a time curve for both analysis techniques (top: dynamics; bottom: sliding average)

The influence of the number of dynamics (control‐label images) is illustrated in Figure 5, where all dynamics before motion correction are shown in the top row, the cumulative perfusion results and the sliding window perfusion results in the bottom row together with time curves for 2 voxels situated in high perfusion areas. Displaying this sliding window average across time gives an impression of the temporal variation of perfusion.

### 3.2 Spatial patterns

**Figure 6:**
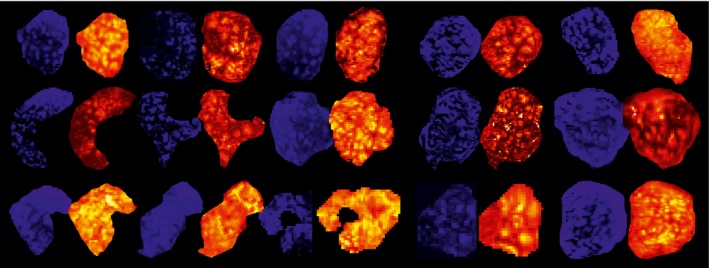
Overview over all 15 PERFOX datasets. $T_{2}^{*}$ and Perfusion maps are given for the central slice. The colormaps are individually scaled from light blue‐dark blue (high‐low perfusion) and light yellow to dark red (high‐low $T_{2}^{*}$)

**Figure 7:**
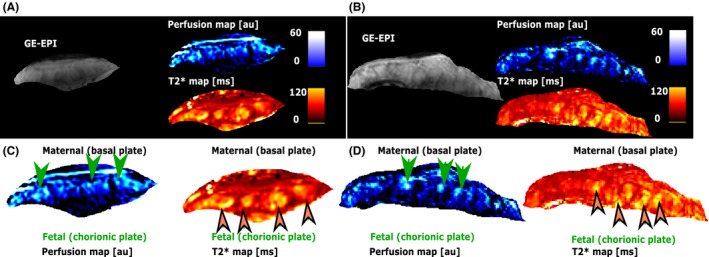
Perfusion and $T_{2}^{*}$ maps obtained from the combined joint PERFOX‐HIGH acquisition, in the axial plane, are shown on exemplary subjects. (A) GA 25+3 and (B) GA 38+1 weeks. The maps are shown separately overlayed on a Gradient‐echo EPI image and then combined with the perfusion map overlayed on the $T_{2}^{*}$ map. in (C,D) a zoom into the placental region is shown, the arrows indicate some of the areas of high $T_{2}^{*}$ and high perfusion within the placental parenchyma

**Figure 8:**
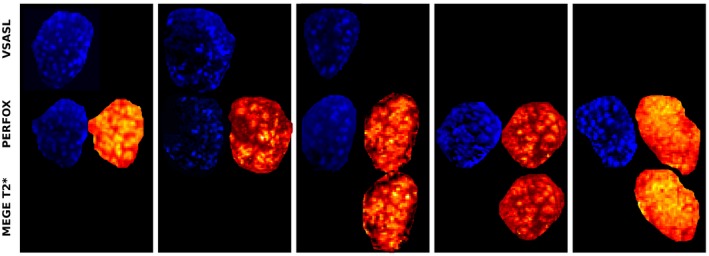
Results from PERFOX scans with matched separate VSASL (top row) and $T_{2}^{*}$ (bottom row) scans. The colormaps are individually scaled from light blue‐dark blue (high‐low perfusion) and light yellow to dark red (high‐low $T_{2}^{*}$)

In the following, $T_{2}^{*}$ results are consistently illustrated with red‐yellow (low‐high) color scale and perfusion results with dark blue—light blue (low‐high) scale. Resulting perfusion and $T_{2}^{*}$ maps from 1 slice are given in Figure 6 for all participants and in Supporting Information Figure S3 for 5 slices of 1 participant. The images illustrate localized regions of high $T_{2}^{*}$ and regions of high perfusion in the coronal planes. While their pattern is similar, the centers of these areas are not spatially co‐localized within each slice. The $T_{2}^{*}$ maps show—in line with previous results^5^, ^19^multiple circular regions of variable size of long $T_{2}^{*}$ with a clear peak in the middle and decay toward the outer regions. Perfusion weighted images and $T_{2}^{*}$ maps are illustrated for the axial high resolution joint PERFOX‐HIGH acquisition in Figure 7 for 2 participants. The perfusion maps illustrate that the areas of highest perfusion appear close to the basal plate. These high perfusion regions then spread out branch‐like from the maternal basal plate toward the chorionic plate. The areas of high $T_{2}^{*}$ are closer to the fetal chorionic plate (see Figure 7C‐D). While only partial coverage of the placenta could be achieved in the transverse scans due to the longest axis of the placenta lying perpendicular to the visualized plane, these scans in this orientation best illustrate the non‐co‐localization of areas of high $T_{2}^{*}$ and perfusion along the maternal‐fetal axis. The results from 3 PERFOX scans, alongside the respective separately acquired VSASL and $T_{2}^{*}$ are shown in Figure 8. They display good qualitative and spatial agreement regarding the location of the high $T_{2}^{*}$ areas for PERFOX and the individual $T_{2}^{*}$ scan. Similarly, the areas of high perfusion areas acquired with PERFOX (middle row) and VSALS (top row) appear to correspond. However, their spatial alignment is less clear.

### 3.3 Parameter evaluation and Quantitative group results

**Figure 9:**
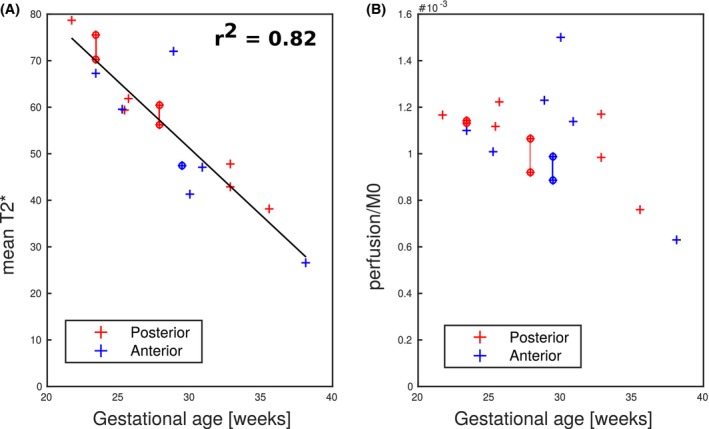
Quantitative results obtained for whole‐placenta ROIs from all PERFOX scans. The mean $T_{2}^{*}$ (A) and the mean perfusion/M0 (B) are depicted. The results from posterior placentas are shown in red, the results from anterior placentas are shown in blue. The repeated scans acquired for 3 subjects are highlighted by circles and connected by a line. The decrease in perfusion with gestational age was not significant

Whole placental ROI analysis was performed on all PERFOX scans. The mean $T_{2}^{*}$ over the whole organ is plotted against gestational age for all 18 scans in Figure 9A, the perfusion/M0 results against gestational age are depicted in Figure 9B. The points are colored by placental location, posterior in red and anterior in blue. $T_{2}^{*}$ and GA are significantly correlated (*F* = 42.43, *P* < 0.05). There is no significant effect of placental location on $T_{2}^{*}$ after controlling for GA (*F* = 0.11, *P* = 0.7426). There is no significant correlation between perfusion/M0 and GA (*F* = 2.18, *P* = 0.1484). There is also no significant effect of placental location on perfusion/M0 after controlling for GA (*F* = 0.07, *P* = 0.7992). The results from the 3 participants with a repeated scan after re‐positioning and new shimming are highlighted by circles and connected by lines. The mean coefficients of variation for these 3 repeated datasets are 4.6±1.5% ($T_{2}^{*}$) and 9.8±6.3% (perfusion).

The multidimensional data obtained with PERFOX allows several further directions of analysis: beside the conventional non‐perfusion weighted $T_{2}^{*}$ maps and the perfusion maps obtained with short TE, perfusion weighted $T_{2}^{*}$ maps and perfusion maps at increasing levels of $T_{2}^{*}$ weighting are shown in Supporting Information Figure S4. The $T_{2}^{*}$ map from the control data shows longer $T_{2}^{*}$ values compared to the tagged $T_{2}^{*}$ map—illustrated as well in the difference map. Analysis of all datasets reveals that the ratio between tagged and control $T_{2}^{*}$, decreases over gestational age (*P* = 0.0057). Similar analysis of the influence of the BGS on the $T_{2}^{*}$ maps did not show statistical significant differences between $T_{2}^{*}$ maps calculated from non‐BGS and BGS volumes (*P* = 0.88).

The joint acquisition also allows to assess the effect of $T_{2}^{*}$ weighting on the perfusion results by calculating the perfusion maps at different $T_{2}^{*}$ weightings. The confounding effect of the $T_{2}^{*}$ contribution to the perfusion map can be completely eliminated by calculating a perfusion map from the proton density maps extracted from $T_{2}^{*}$ fitting. Example results comparing the perfusion map from the 1st TE and the proton density maps are shown in Supporting Information Figure S5. They are in good agreement for the central slices, but reveal differences which are localized mainly in the regions between the functional lobules.

## 4 DISCUSSION

This study presents a combined sequence for placental perfusion and oxygenation (PERFOX) measurement with required essential post‐processing, mainly motion correction and quantification on 15 pregnant women. PERFOX is the first example of application of this dual‐contrast acquisition to the placenta. A key advantage is the higher consistency within the multimodal dataset, compared to a separate/sequential acquisition as well as the ability to dynamically resolve both essential quantities jointly. ASL measurements have, however, been previously combined with T_2_ 32, 33 and diffusion measurements.^34^, ^35^ The combination with $T_{2}^{*}$ was originally exploited combining data from separate acquisitions at multiple echo times^36^ and then extended to dual‐echo acquisitions, mainly for simultaneous measurements of cerebral blood flow and BOLD in fMRI.^37^, ^39^

The simultaneous acquisition allows to observe a clear spatial pattern in all our participants, with areas of high‐perfusion centered close to the maternal basal plate and areas of high $T_{2}^{*}$ closer to the fetal chorionic plate. This shift can be best observed in transverse scanning plane due to the curved geometry of the placenta with regard to the main imaging planes. Furthermore, the low $T_{2}^{*}$ on the in‐flowing highly oxygenated maternal blood (identified by high perfusion signal) compared to the mid‐parenchymal high‐ $T_{2}^{*}$ peaks might indicate that these observed high $T_{2}^{*}$ regions are linked not only to oxygenation state but to either blood flow velocity, exchange or properties of fetal hemoglobin. The exact physiological pathway resulting in this observed behavior remains however unclear, but dynamic multi‐contrast techniques such as the acquisition presented here might help to shed light on these processes.

The analysis provided here, showed that the number of required dynamics for stable perfusion signal in the placenta is in the range of 5‐8 for the chosen acquisition parameters. This allows either to reduce the number of dynamics and thus limit the required acquisition time, or a dynamic assessment using a sliding window analysis as illustrated in Figure 5.

The high contrast to noise ratio resulting from high perfusion values observed in the placenta are essential for this. These observations will not directly translate to less blood‐rich organs such as the brain, where a higher number of dynamics is required for robust perfusion visualization, resulting in coarser temporal resolution.

The observed strong linear decrease over gestation in $T_{2}^{*}$ and weak negative correlation between perfusion and gestation are in line with those observed separately in previous studies.^32^, ^33^ Zun et al^16^ reported higher perfusion for posterior placentas. Our data show no such significant difference based on placental location. There is little evidence of differences in placental function between anterior and posterior placental locations in literature, and our results would support this. The low number of participants in the current placental ASL studies however call for caution regarding both differences with location and trends over gestation.

One prior study showed differences in perfusion between lateral and supine positioning.^16^ In contrast, at our institution all pregnant participants are scanned in supine position while under constant monitoring and splitting the scanning time.

Recent placental ASL studies used 3D readouts^39^ in contrast to the 2D EPI acquisition chosen for this study. We selected 2D EPI due to its ability to freeze motion within each slice, the flexibility to optimize the echo spacing in order to reduce acoustic noise and to acquire the data required for $T_{2}^{*}$ relaxometry with multiple echoes in quick succession.

Compared with more conventional separate acquisitions which allow individual optimization of sequence parameters for each modality—e.g. 4 echo times spanning a wide range for $T_{2}^{*}$ mapping and narrow range of PLDs for all slices—the joint acquisition inevitably forces compromises on these constraints. Therefore, i.e. only 3 echo times for $T_{2}^{*}$ mapping with lower maximal TE of 93 ms (instead of 148ms) and a reduced coverage of 8 slices (instead of 13) were chosen to keep the acquisition time for all slices at all 3 echo times as compact as possible. Nevertheless, the obtained functional maps illustrate usable data of good quality. The Rician noise distribution in conventional MRI images, approaching Gaussian distribution only for high signal‐to‐noise ratio (SNR), is important to consider for the $T_{2}^{*}$ fits. Low SNR is associated with longer echo times, which result, e.g. from longer EPI trains to achieve higher resolution multi‐echo $T_{2}^{*}$ scans or if more than 4 echoes are acquired for multi‐exponential multi‐compartment fits. However, in our case, the requirement to reduce PLD resulted in only 3 echo times acquired at comparably low resolution of 3 mm. The short TEs ensure robust mono‐exponential fitting, but do, however, not support higher order fitting.

VSASL often employs a second velocity selective module immediately before the acquisition (this sequence is referred to as dual VSASL or DVASL in^28^): it saturates blood flowing above the cutoff velocity and thus acts as a filter to make sure signal contributions from blood accelerating during the PLD are eliminated (i.e. in the brain this would be venous blood); without this second module VSASL images are very difficult to quantify as they have contributions from both arterial and venous flow.^28^ However, for this study VSASL with a single VS module was chosen for a number of reasons. Firstly, the complex placental vasculature with slow‐flowing oxygen‐rich blood between the villi and oxygen‐poorer and higher‐velocity venous backflow does not allow a clear velocity‐based separation into arterial and venous blood. Secondly, each of the encoding modules leads to substantial T_2_‐dependent signal decay. With our 50ms‐long module and assuming T_2_ of blood to be roughly 170 ms, we have a 35.5% signal loss/module. Less oxygenated blood with lower T_2_ will experience an even higher signal reduction. Finally, the stricter specific absorption rate (SAR) limitation for fetal studies puts a time penalty on the DVSASL technique where 2 additional 90‐degree pulses plus an adiabatic refocusing pulse are required. In our case adding the 2nd module raised the minimally achievable repetition time from 3.5 s to 6.4 s. This would thus either increase the acquisition time or decrease the number of acquired label‐control pairs. Evaluation of dual VSASL vs. the protocol used in this study is underway.

This study does not describe variations of the velocity encoding direction; head to feet encoding was chosen for all scans irrespective of scan orientation, similar to.^16^ The results reported appear to be consistent with labeling of maternal blood. Experiments are ongoing to further quantify the effect of velocity encoding in different directions. One of the previous placental VSASL studies^17^ reported a within‐subject coefficient of variation of only 3.5% on whole‐placenta perfusion values. While the coefficient of variation reported here is higher at 9.8%, it is important to note that we assessed reproducibility between 2 sessions, providing a much more appropriate estimate of data reliability for a clinically useful scanning scenario than the back‐to‐back scanning reported in.^17^

Six separate conventional VSASL and $T_{2}^{*}$ scans were acquired in a subgroup of subjects. However, whilst the $T_{2}^{*}$ scans were acquired immediately before or after the PERFOX scans, within the same session, the separate perfusion scans were acquired in different scanning sessions due to the restrictions on continuous scanning time for pregnant women. This difference is reflected both qualitatively and quantitatively: while both show visually good agreement regarding the location and size of the areas of high perfusion and $T_{2}^{*}$ , it was significantly harder to find the same geometrical location for the perfusion maps acquired in different scanning sessions due to changes in maternal positioning, fetal lie, location of the fundus and planning of the region of interest for the acquisition. Future work will include validation with a static perfusion phantom.

In this study, the joint‐acquisition data was presented separately by processing along the echo times for relaxometry and in a separate step along dynamics for perfusion information. However, the data can be processed to show differences in the $T_{2}^{*}$ maps calculated from control and labeled volumes; the possibility to calculate the perfusion map from the proton density map—allowing to correct for $T_{2}^{*}$ effects—is also appealing. Both are additional benefits of the joint acquisition. Indeed, the data are ideally suited for a fully combined analysis approach, that will be explored in the future.

This study proposes a novel combined strategy to obtain the co‐localized functional descriptor visualizing perfusion and oxygenation. The PERFOX joint acquisition will be used in the future to study a variety of functional mechanisms such as the causality of insufficiency in pregnancy complications like preeclampsia, growth restriction, and congenital heart disease. Visualizing an imbalance between perfusion and oxygen uptake might allow to explore compensatory mechanisms and to study variations over placental surface and thus possibly deviations in implantation. This might ultimately benefit the deeper understanding of placental physiology and disease etiology. Lastly, the proposed joint PERFOX sequence might furthermore find applications in other, highly perfused organ systems such as kidney or liver where an equal interest exists with regard to separating perfusion and oxygenation effects.

## Supporting information


**FIGURE S1** Post‐labeling delays illustrated for all slices and echoes for both ‘separate’ VSASL with 13 slices (blue) and the 2 PERFOX variants with 8 slices used in the paper (standard PERFOX in green and PERFOX‐HIGH in red). The first echo is marked by a large star, second and third echoes by smaller stars
**FIGURE S2** Illustration of the influence of the different scan orientations on the acquired signal. (A) Schematic illustration of the 2 scan orientations employed in this paper—coronal and transverse. (B‐C) Control images from the coronal (red background) and transverse (green background) acquisitions, each is also displayed reformatted in the non‐native orientation. (B) displays the results without and (C) with background suppression. Finally, (D) displays a zoom into both acquisitions together with yellow arrows to illustrate the direction of increasing PLD
**FIGURE S3** Results from a coronal PERFOX scan at GA 29+1 weeks. Five consecutive slices are shown for the anatomical GE‐EPI volume (first row), the perfusion maps (second row) and the $T_{2}^{*}$ maps (third row)
**FIGURE S4** A, $T_{2}^{*}$ maps calculated from the control volumes, labeled volumes and difference in $T_{2}^{*}$ between the 2. B, Perfusion maps at the 3 different echo times TE acquired in PERFOX. C, Evaluation over all subjects of the mean $T_{2}^{*}$ values from tagged and control volumes relative to the mean $T_{2}^{*}$ from control volumes
**FIGURE S5** Perfusion maps obtained from the proton density maps and from the data from the 1st echo time together with difference image
