# Supplementary material for: Perfusion and apparent oxygenation in the human placenta (PERFOX)
Source: Magn Reson Med. 2019 Aug 21;83(2):549–60. doi: 10.1002/mrm.27950 (PMC6825519; doi:10.1002/mrm.27950)
Supplement: Supplementary file 1 — FIGURE S1 Post‐labeling delays illustrated for all slices and echoes for both ‘separate’ VSASL with 13 slices (blue) and the 2 PERFOX variants with 8 slices used in the paper (standard PERFOX in green and PERFOX‐HIGH in red). The first echo is marked by a large star, second and third echoes by smaller stars FIGURE S2 Illustration of the influence of the different scan orientations on the acquired signal. (A) Schematic illustration of the 2 scan orientations employed in this paper—coronal and transverse. (B‐C) Control images from the coronal (red background) and transverse (green background) acquisitions, each is also displayed reformatted in the non‐native orientation. (B) displays the results without and (C) with background suppression. Finally, (D) displays a zoom into both acquisitions together with yellow arrows to illustrate the direction of increasing PLD FIGURE S3 Results from a coronal PERFOX scan at GA 29+1 weeks. Five consecutive slices are shown for the anatomical GE‐EPI volume (first row), the perfusion maps (second row) and the T2* maps (third row) FIGURE S4 A, T2* maps calculated from the control volumes, labeled volumes and difference in T2* between the 2. B, Perfusion maps at the 3 different echo times TE acquired in PERFOX. C, Evaluation over all subjects of the mean T2* values from tagged and control volumes relative to the mean T2* from control volumes FIGURE S5 Perfusion maps obtained from the proton density maps and from the data from the 1st echo time together with difference image [file MRM-83-549-s001.pdf]

ORIGINAL ARTICLE

Journal Section

Supporting Information for: Perfusion and apparent oxygenation in the human placenta (PERFOX)

Jana Hutter[1] | Anita A. Harteveld[2] | Laurence H. Jackson[1] | Suzanne Franklin[3] | Clemens Bos[2] | Matthias J. P. van Osch[3] | Jonathan O'Muircheartaigh[1] | Alison Ho[4] | Lucy Chappell[4] | Joseph V Hajnal[1] | Mary Rutherford[1] | Enrico De Vita[1]

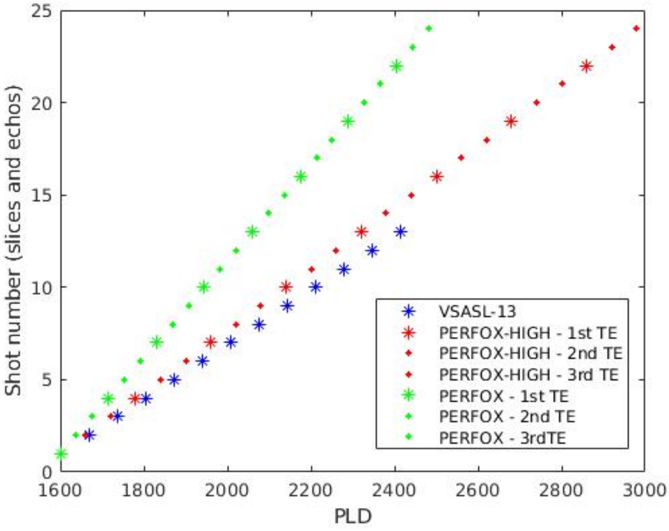

Supporting Information Figure S1: Post labeling delays illustrated for all slices and echoes for both 'separate' VSASL with 13 slices (blue)) and the two PERFOX variants with 8 slices used in the paper (standard PERFOX in green and PERFOX-HIGH in red). The first echo is marked by a large star, second and third echoes by smaller stars.

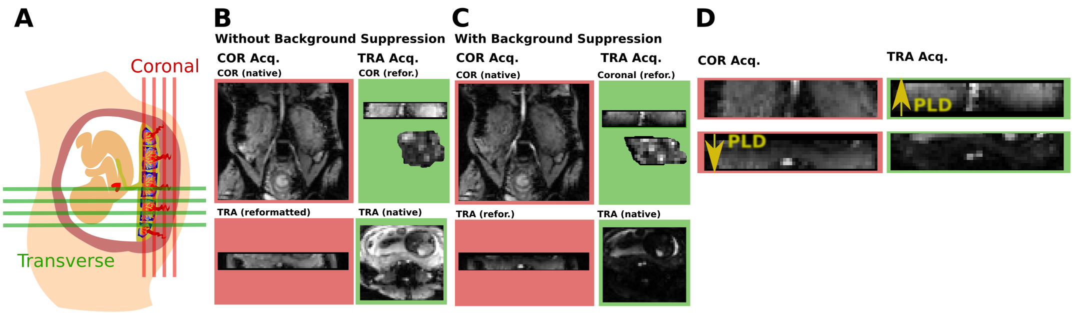

Supporting Information Figure S2: Illustration of the influence of the different scan orientations on the acquired signal. (a) Schematic illustration of the two scan orientations employed in this paper - coronal and transverse. (b-c) Control images from the coronal (red background) and transverse (green background) acquisitions, each is also displayed reformatted in the non-native orientation. (b) displays the results without and (c) with background suppression. Finally, (d) displays a zoom into both acquisitions together with yellow arrows to illustrate the direction of increasing PLD.

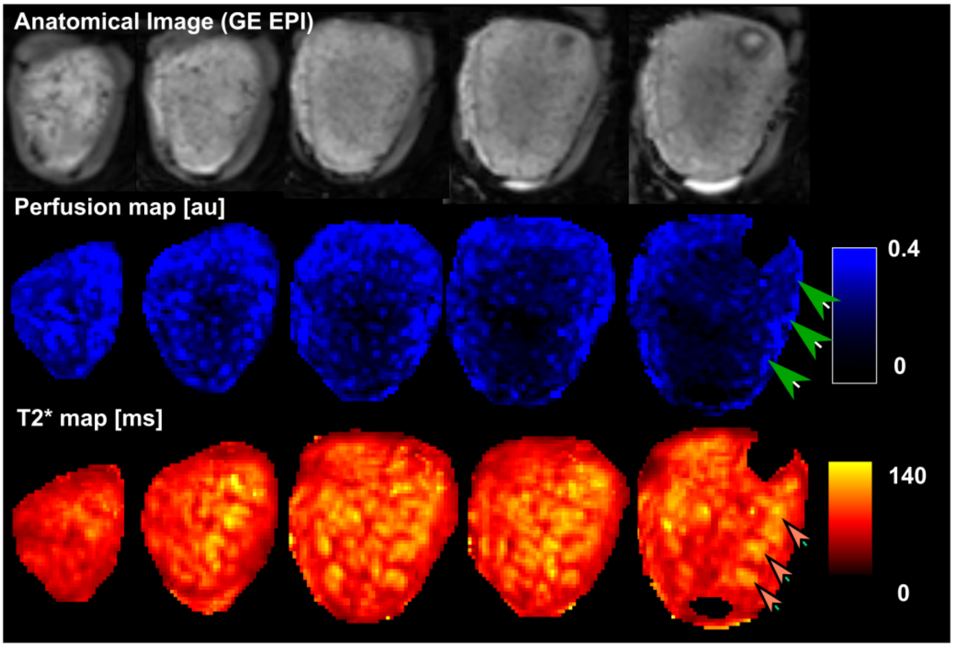

Supporting Information Figure S3: Results from a coronal PERFOX scan at GA 29+1 weeks. Five consecutive slices are shown for the anatomical GE-EPI volume (first row), the perfusion maps (second row) and the T2* maps (third row). The arrows in the last column illustrate the different distribution of high perfusion areas closer to the maternal basal plate (green arrows) and the areas of high T2* closer to the fetal chorionic plate (red arrows).

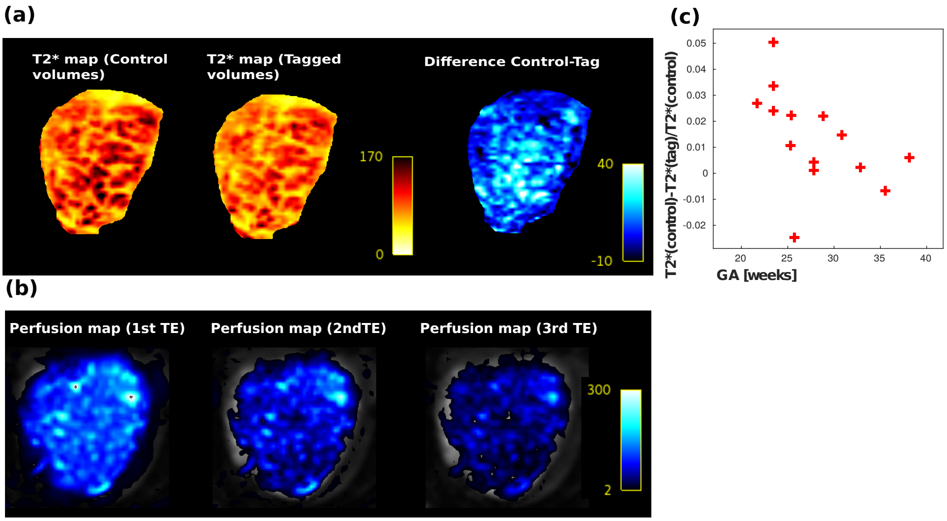

Supporting Information Figure S4: (a) T2* maps calculated from the control volumes, labelled volumes and difference in T2* between the two.(b) Perfusion maps at the 3 different echo times TE acquired in PERFOX. (c) Evaluation over all subjects of the mean T2* values from tagged and control volumes relative to the mean T2* from control volumes.

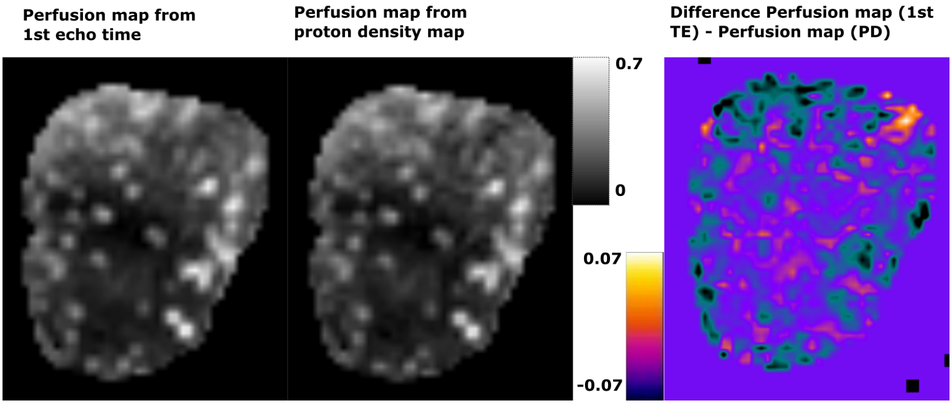

Supporting Information Figure S5: Perfusion maps obtained from the proton density maps and from the data from the 1st echo time together with difference image.
